# A Case of Hemolytic Disease of the Newborn due to Di^**a**^ Antibody

**DOI:** 10.1155/2015/897803

**Published:** 2015-11-22

**Authors:** Ashif Jethava, Esperanza Olivares, Sherry Shariatmadar

**Affiliations:** Department of Pathology, University of Miami, Jackson Health System, Miami, FL, USA

## Abstract

Anti-Di^a^ is a clinically significant red cell antibody known to cause hemolytic disease of the newborn. Here, we report on a case of mild hemolytic disease of the newborn caused by Di^a^ antibody. The mother had three prior pregnancies with no history of blood transfusion. She delivered a preterm 35-week-old female newborn by cesarean section. The neonate developed anemia and mild icterus on postnatal day five with hemoglobin of 9500 mg/dL and total bilirubin of 10 mg/dL. The direct antiglobulin test on the neonate's red blood cells was positive. The maternal serum and an eluate from the infant RBCs were negative in routine antibody detection tests but were positive using commercially prepared Di(a+) red cells. The neonate was discharged home in stable condition following treatment with erythropoietin and phototherapy. When a newborn has a positive DAT in the absence of major blood group incompatibility or commonly detected RBC antibodies, an antibody to a low frequency antigen such as Di^a^ must be considered. Further immunohematology tests are required to determine presence of the antibody and the clinician must be alerted to closely monitor the infant for signs of anemia and hemolysis.

## 1. Introduction

The first antigen assigned to the Diego blood group system, Di^a^, was described by Layrisse et al. in 1955 [1]. They reported an antibody to a low frequency antigen in the serum of a Venezuelan woman (Mrs. Diego) which caused fatal hemolytic disease of the newborn (HDN). The existence of the antibody had been noted briefly in another report one year earlier [2]. The prevalence of the Di^a^ antigen is known to be different among races, which has made the Diego blood group attractive to anthropologists [3]. It is very rare among Caucasians and Blacks (0.01%) but relatively common among the South American Indians (36%) and Asians of Mongoloid origin (5–15%) which includes the Japanese, Chinese, and Koreans [4–8]. Anti-Di^a^ has been reported to cause moderate to severe HDN [9–14] and rarely a hemolytic transfusion reaction [15]. Here we report a case of HDN caused by Di^a^ antibody. The newborn developed anemia and moderate hyperbilirubinemia which required erythropoietin injection and phototherapy.

## 2. Case Presentation

A 30-year-old South American woman, G4P3L3, with a history of preterm labor, placenta previa, and cesarean section × 3 and no prior history of transfusions gave birth to a preterm 35-week-old female newborn by cesarean section. Records of her antenatal care were not available to us as she presented to our hospital for the first time following arrival from Peru. The newborn infant had a birth weight of 2,900 grams with an Apgar score of 8. Soon after birth, the neonate was noted to have an episode of respiratory distress and drop in oxygen saturation to 82% requiring frequent suctioning and continuous oxygen support. She was admitted to the neonatal intensive care unit for further evaluation and monitoring. Initial chest X-ray demonstrated bilateral perihilar and lower lobe interstitial infiltrates for which she was started on broad spectrum intravenous ampicillin and gentamycin antibiotics. Blood culture, urinalysis, and urine for microscopic examination were ordered and reported as negative. On the fifth day, the neonate was noted to be pale and icteric with clinical signs of anemia. Laboratory findings were as follows: RBC 2.71 × 10^6^  cells/mcl; white blood cell count 11.7 × 10^9^/L; hemoglobin 9.5 mg/dL; hematocrit 26.5%; reticulocyte count 6.5%; platelet count 435 × 10^9^/L; and liver function test showed a total bilirubin of 10 mg/dL with predominance of unconjugated hyperbilirubinemia. Extensive investigation was performed to determine the cause of anemia and hemolysis which included tests for cord blood glucose-6-phosphate dehydrogenase (G6PD) and parvovirus B19, both of which were negative.

Immunohematology workup revealed that both the mother and the infant were blood group O, RhD positive. Direct antiglobulin test (DAT) was ordered on the neonate's and mother's red blood cells. It was weakly positive (1+) with monospecific anti-human globulin (AHG) IgG on the neonate's RBCs and negative on the mother's RBCs. The maternal serum and an eluate prepared from neonate's red blood cells showed negative reactions in routine antibody detection tests, but after testing with cells of rare phenotypes, they demonstrated an alloantibody reacting with the Di(a+) red cells by indirect antiglobulin test (IAT) in the AHG phase.

The neonate was successfully treated with subcutaneous erythropoietin injection three times for a week, followed by intensive phototherapy. The bilirubin level dropped to 6.7 mg/dL within few days of treatment. The infant was discharged home in good clinical condition with the following laboratory findings: RBC 3.18 × 10^6^cell/*μ*L; hemoglobin 11.2 mg/dL; hematocrit 32.3%; and a reticulocyte count of 2.5%.

## 3. Methods

Postnatal screening for unexpected RBC antibodies was performed using tube methodology including Low Ionic Strength Solution (LISS) (Clinical Diagnostics, Raritan, NJ) and polyethylene glycol (PeG) techniques (Immucor Inc., Norcross, GA, USA) with commercially prepared screening cells (Medion Grifols Diagnostics AG, Switzerland) at 37°C and indirect antiglobulin test (IAT) according to the manufacturer's instructions. The DAT was performed using the tube methodology with poly- and monospecific IgG anti-human globulin (Bio-Rad Medical Diagnostics, Dreieich, Germany). An antibody elution was performed on the neonate's DAT positive RBCs obtained by acid elution with use of commercial reagents (Gamma ELU-KIT, Immucor, Rodemark, Germany).

The neonate's eluate was tested against two screen cells (Medion Grifols Diagnostics AG, Switzerland) and a panel of six reagent RBCs (three Di(a+) RBCs were included). Maternal serum was tested against three selected cells positive for Di^a^ antigen at room temperature, 37°C, and IAT phase (Panocell-20, Immucor, Norcross GA, USA).

Determination of RBC antibody specificities in the mother's serum and of the neonate's RBC eluate using commercial RBC panels produced 2+ reactions solely with three Di(a+) test RBCs.

## 4. Discussion

The Diego blood group system currently consists of 22 antigens, including three pairs of antithetical antigens: Di^a^/Di^b^, Wr^a^/Wr^b^, and WU/DISK. The antigens are located on the red blood cell membrane transporter also known as Band 3, encoded by a SLC4A1 gene on chromosome 17q12-q21 [16]. Band 3 acts as an anion exchanger between chloride and bicarbonate ions which helps transport carbon dioxide from the tissues to the lung. It also helps to maintain the structural integrity of the red blood cell membrane by stabilizing membrane lipids [16–18]. The Di^a^ antigen is fully developed on the red cells of the newborn infants as it is on the red cells of adults. Di^a^ antibodies are polyclonal IgGs of subclasses IgG1 and IgG3. These antibodies occasionally bind complement and lyses untreated red blood cells [16–18].

Based on genetic studies, there is great variation in the distribution of the Di^a^ antigen in different races. It is relatively common among the South American Indians and Asian of Mongolian origin and rare in Caucasian and Blacks [5–8]. Because of the different prevalence of the Di^a^ antigen, it has been of great interest to the field of anthropology and transfusion medicine. Anti-Di^a^ is known to be dangerous to the fetus and newborn and has been associated with moderate to severe HDN, at least one of which was fatal [9–14].

Monestier et al. described a woman who gave birth to an infant with hyperbilirubinemia and a strongly positive DAT. During the pregnancy and after delivery, the mother had negative RBC antibody screening tests using standard red blood cell panels, but the indirect antiglobulin test between the mother's serum and the father's red blood cells was strongly positive. The antibody was eluted from the newborn infant's red blood cells and was identified as anti-Di^a^ [14]. Hundric-Haspl reported anemia in a 3-week-old infant who had been discharged home in good condition shortly after birth. The infants DAT was positive shortly after birth while testing of the mother's serum and the eluate of infants RBCs using routine immunohematology tests were negative. Repeat testing following readmission showed similar results on routine tests; however, on testing with extensive panel of red blood cells, anti-Di^a^ was identified in the mother's serum and the eluate of the infants RBCs [19]. In our case, postpartum antibody screening test of the mother's plasma against common red cell antigens was negative. The newborn initially had no clinical signs of HDN in spite of a positive DAT. Anemia and moderate hyperbilirubinemia were noted five days after birth and successfully treated with phototherapy and erythropoietin injection. The maternal plasma and infant's eluate from red blood cells were negative in routine antibody detection tests but were positive when tested against Di(a+) RBCs.

Development of Di^a^ antibodies mostly occurs following alloimmunization of a woman who is negative for the Di^a^ antigen while carrying a fetus who has inherited the antigen from the father [9–14, 19]. The usual immunizing event is delivery and fetomaternal hemorrhage is more commonly encountered with C-section delivery [20]. Immunization can also occur following trauma, amniocentesis, cordocentesis, abortion, or other procedures. In majority of the cases, the presence of Di^a^ antibodies in the plasma of an immunized pregnant woman cannot be determined by routine screening test, as most antibody screening cells lack the Di^a^ antigen. Therefore, there may be no serologic evidence that HDN is present and even with standard prenatal care, the diagnosis may not be apparent until after delivery, when the newborn is found to have a positive DAT and clinical signs of HDN. Because of the low frequency of the antigen in our population, finding compatible blood is not difficult if the neonate needs a blood transfusion.

## 5. Conclusion

Whenever a newborn has a positive DAT result in the absence of major blood group incompatibility or reactivity with common red cell antigens, it is important to consider that the positive DAT may be due to alloimmunization to a low frequency RBC antigen such as Di^a^ that cannot be detected in routine antibody detection tests. Further testing with rare RBC antigens should be performed to determine the presence of the antibody and the infant should be closely monitored and treated for clinical signs of anemia and hemolysis.

## References

[1] Layrisse M., Arends T., Dominguez S. R. (1955). Nuevo grupo sanguineo encontrado en descendientes de Indios. *Acta Médica Venezolana*.

[2] Levine P., Koch E. A., McGee R. T., Hill G. H. (1954). Rare human isoagglutinins and their identification. *American Journal of Clinical Pathology*.

[3] Layrisse M. (1958). Anthropological considerations of the Diego (Di^a^) antigen. Possible application in the studies of Mongoloid and hybrid populations. *American Journal of Physical Anthropology*.

[4] Simmons R. T., Albrey J. A., Morgan J. A. G., e tal (1968). The Diego blood group: anti—Di^a^ and Di (a+) blood group antigen found in Caucasians. *Medical Journal of Australia*.

[5] Layrisse M., Arends T. (1957). The Diego blood factor in Negroid populations. *Nature*.

[6] Junqueira P. C., Wishart P. J., Ottensooser F., Pasqualin R., Fernandez P. L., Kalmus H. (1956). The Diego blood factor in Brazilian Indians. *Nature*.

[7] Junqueira P. C., Wishart P. J., Ottensooser F., Pasqualin R., Fernandez P. L., Kalmus H. (1956). The *Diego* blood factor in Brazilian Indians. *Nature*.

[8] Layrisse M., Arends T. (1956). The *Diego* blood factor in Chinese and Japanese. *Nature*.

[9] Levine P., Robinson E. A., Layrisse M., Arends T., Sisco R. D. (1956). The Diego blood factor. *Nature*.

[10] Alves De Lima L. M., Berthier M. E., Sad W. E. (1982). Characterization of an anti-Dia antibody causing hemolytic disease in a newborn infant. *Transfusion*.

[11] Kusnierz-Alejska G., Bochenek S. (1992). Hemolytic disease of the newborn due to anti-Di^a^ and the incidence of Di^a^ antigen in Poland. *Vox Sanguinis*.

[12] Chung M. A., Park E. H., Lee C. H. (2002). A case of hemolytic disease of the newborn due to anti Di^a^ antibody. *Journal of the Korean Society of Neonatology*.

[13] Ting J. Y., Ma E. S., Wong K. Y. (2004). A case of severe haemolytic disease of the newborn due to anti-Di(a) antibody. *Hong Kong Medical Journal*.

[14] Monestier M., Rigal D., Meyer F. (1984). HDN caused by anti Di^a^ antibodies. *Archives Françaises de Pédiatrie*.

[15] Hinckley M. E., Huestis D. W. (1979). An immediate hemolytic transfusion reaction apparently caused by anti-Di^a^. *Revue Française de Transfusion et Immuno-Hématologie*.

[16] Figueroa D. (2013). The Diego blood group system: a review. *Immunohematology*.

[17] Issitt P. D., Anstee D. J. (1998). *Applied Blood Group Serology*.

[18] Fung M. K., Hillyer C. D., Grossman B. J., Westhoff C. M. (2014). *Technical Manual*.

[19] Hundric-Haspl Z., Balen-Marunic S., Tomasic-Susanj E., Tomicic M., Vujaklija-Stipanovic K. (2003). Anti-Diego^a^ red blood cell alloantibody as a possible cause of anemia in a 3-week-old infant. *Archives of Medical Research*.

[20] Sebring E. S., Polesky H. F. (1990). Fetomaternal hemorrhage: incidence, risk factors, time of occurrence, and clinical effects. *Transfusion*.

